# Effect of Temperature and Pyrolysis Atmosphere on Pore Structure of Sintered Coal Gangue Ceramsites

**DOI:** 10.3390/ma18143386

**Published:** 2025-07-18

**Authors:** Baoqiang Zhao, Xiangjie Duan, Yu Li

**Affiliations:** 1 State Key Laboratory of Advanced Metallurgy, University of Science and Technology Beijing, Beijing 100083, China; zbq0888@163.com (B.Z.); duanxiangjie@gmail.com (X.D.); 2 School of Chemistry and Chemical Engineering, Harbin Institute of Technology, Harbin 150001, China

**Keywords:** coal gangue ceramsites, pore structure, temperature regimes, phase transition, pyrolysis atmosphere

## Abstract

The sintering of coal gangue ceramsites (CGCs) using belt roasting technology involves the recirculation of flue gases and variations in oxygen concentrations. This study investigates the effects of temperature and pyrolysis atmosphere on the pore structure of CGCs at three temperature levels: 600 °C, 950 °C, and 1160 °C. The results revealed that apparent porosity is primarily influenced by O_2_-promoted weight loss and the densification process, while closed porosity is affected by pyrolysis reactions and crystal phase transformations. Below 950 °C, enhancing the oxidative atmosphere facilitates the preparation of porous CGCs, whereas above 950 °C, reducing the oxidative atmosphere favors the preparation of high-strength CGCs. These findings provide valuable insights for the industrial production of CGCs, offering a basis for optimizing sintering parameters to achieve the desired material properties. The latest production equipment, furnished with adjustable atmospheres (such as belt sintering roasters), can better regulate the mechanical properties of the products.

## 1. Introduction

The utilization of coal gangue (CG), a significant industrial byproduct, has garnered considerable attention due to its environmental and economic benefits [1]. In China, coal gangue is mainly concentrated in regions such as Shanxi, Yulin of Shaanxi, Ordos of Inner Mongolia, and Xinjiang. To date, the cumulative stockpiles of coal gangue in China have exceeded 7 billion tons [2]. Such a large stockpile has led to a waste of resources, as well as serious environmental and safety hazards. Among the various applications, the production of coal gangue ceramsites (CGCs) through sintering processes offers a promising pathway for large-scale valorization [3]. Ceramsite, a lightweight aggregate, is widely used in construction [4], agriculture [5], wastewater treatment [6], and other applications due to its desirable properties, such as its high strength, low density, and excellent thermal insulation.

Previous research has reported on the production of ceramsite using a sintering machine to simulate the belt roasting process [7]. It was observed that the sintering process of CGCs using belt roasting technology is characterized by flue gas recirculation and fluctuations in oxygen concentrations. These findings align with the results of Abzalov et al. [8]; in the industrial production process, by scientifically and rationally setting the roasting system and performing targeted optimizations of the design of the gas circulation system, it is possible not only to maximize the recovery and utilization of heat contained in exhaust gases—thereby providing strong guarantees for products to meet the expected performance standards—but also to practically achieve the goal of reducing energy consumption on this basis. This approach thus ensures the product quality while enhancing the economic efficiency of the production. Such variations in temperature and atmosphere significantly impact the microstructure and properties of the final product. Understanding these effects is crucial for optimizing the production process and enhancing the quality of CGCs.

While the majority of research has primarily focused on the macroscopic properties of ceramsite, Xiaolin Pan et al. [9] investigated the fabrication of ultra-lightweight ceramsite from high-calcium red mud, elucidating its physical characteristics and expansion mechanisms. Yifan Liu et al. [10] described contemporary ceramsite production methods and pore formation mechanisms in porous ceramsite but did not examine how pores affect physical performance. Currently, studies specifically exploring the effect of pore structure on ceramsite properties are limited. However, pore structure—including apparent and closed porosity—plays a crucial role in shaping the mechanical properties, water absorption, and other functional features [11].

As coal gangue generally contains residual coal, the combustion of the coal during the sintering process of CGCs is beneficial for saving heating energy consumption, but it also results in a more complex CGC pore structure.

The evolution of the pore structure and the sintering characteristics of CGCs during the roasting process have been analyzed [12]. This analysis revealed two critical temperatures, 950 °C and 1160 °C, where significant changes in porosity occur. Below 950 °C, the combustion of carbon and other combustible components in the ceramsite leads to the formation of an initial pore structure, with true porosity, water absorption, apparent porosity, and closed porosity maintained at approximately 45%, 20%, 32%, and 12%, respectively, and compressive strength below 2 MPa. Between 950 °C and 1160 °C, the true porosity and apparent porosity decrease, while the closed porosity and compressive strength increase. Above 1160 °C, the performance of ceramsite deteriorates.

Based on these findings, it is proposed that approximately 950 °C is optimal for producing porous CGCs with high porosity and moisture retention, whereas 1100–1160 °C is suitable for producing high-strength dense CGCs.

Building on previous research, this study focuses on the interactions between temperature, oxygen concentration, and pore structure development during the sintering process. Experiments were conducted at three different temperatures: 600 °C, 950 °C, and 1160 °C, and at oxygen concentration levels of 15% and 20%. The results of this study will provide theoretical parameters for the industrial production of coal gangue. Through the rational design of the flue gas circulation pipeline in the industrial production line of coal gangue ceramsite, it will offer theoretical support for the production of different types of coal gangue ceramsite. Meanwhile, it will promote the sustainable and efficient utilization of industrial wastes.

## 2. Materials and Methods

### 2.1. Coal Gangue (CG)

CG samples were collected from Erdos City, Inner Mongolia, China. The chemical composition of the CG powders at room temperature (25 °C) was determined using X-ray fluorescence (XRF, Rigaku ZSX Primus, Rigaku Corporation, Tokyo, Japan). Additionally, the chemical composition of the CGCs after sintering at 1150 °C is presented in Table 1, indicating minimal elemental changes before and after sintering. The proximate analysis and calorific value of CG are provided in Table 2. The procedure for determining the loss on ignition (LOI) is as follows: First, the sample is ground to a particle size of ≤75 μm. Two crucibles, which have been calcined at 1050 °C and cooled, are prepared, and the mass of each empty crucible (m_0_) is weighed. Next, 1–5 g of the sample is added to each crucible, and the total mass of the crucible and the sample (m_1_) is weighed. The crucibles are placed in a muffle furnace, with the temperature gradually increased to 1050 °C and maintained for 2–4 h. After the temperature drops below 200 °C, the crucibles are taken out and cooled, and the total mass of the crucible and the residual (m_2_) is weighed. The LOI is calculated using the following formula: (m_1_ − m_2_)/(m_1_ − m_0_) × 100%. The measurement is performed in duplicate, and the average value is taken.

### 2.2. Preparation of Coal Gangue Ceramsites (CGCs)

Experiments were conducted at 600 °C, 950 °C, and 1160 °C using a tube furnace (Chengyi T1260A, Henan Chengyi Laboratory Equipment Co., Ltd., Zhengzhou, China). The study aimed to investigate the effects of temperature and oxygen concentrations (15% and 20%) on the pore structure of CGCs. The conditions are labeled as “Temperature&time” (°C and min). For example, “600&60” denotes 600 °C and 60 min with 20% O_2_, while “600&60LO” denotes 600 °C and 60 min with 15% O_2_. During the pyrolysis process, the total gas flow rate was kept constant. By adjusting the flow rates of N_2_ and O_2_ using flow meters, mixed gases with different O_2_ concentrations (15% and 20%) were produced.

Experimental Design:

600 °C: Dried CGCs were purged with N_2_ at 30 mL/min for 5 min, then heated to 600 °C at 10 °C/min under N_2_. Different O_2_ concentrations were achieved by adjusting the N_2_ and O_2_ flow rates. After holding, the furnace was cooled under N_2_. Labeled as “600&30,” “600&60,” “600&60LO,” and “600&120.”

950 °C: Following the 600 °C holding period, the CGCs were heated to 950 °C at 10 °C/min under N_2_. Labeled as “600&30 + 950&30,” “600&30 + 950&60,” “600&60 + 950&30,” and “600&60 + 950&30LO.”

1160 °C: Following the 950 °C holding period, the CGCs were heated to 1160 °C at 10 °C/min under N_2_. Labeled as “600&30 + 950&30 + 1160&60,” “600&60 + 950&30 + 1160&30,” “600&60 + 950&30 + 1160&30LO,” and “600&60 + 950&30 + 1160&60.”

**Figure 1:**
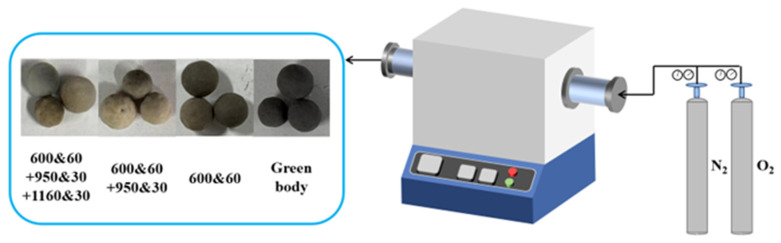
The experimental setup and ceramsite samples at different temperatures (25, 600, 950, and 1160 °C).

The experimental setup and ceramsite samples are shown in Figure 1.

### 2.3. Characterization Methods

The true, apparent, and closed porosity, volume density, and water absorption were tested using a density tester (DT, Vicometer WKT-300C, Vicometer Co., Ltd., Taizhou, China), and 10 CGCs were simultaneously measured for each set of results. A simultaneous thermal analyzer with a Type S thermocouple (TA, STA 449 F3, Netzsch, Selb, Germany) was used to measure the mass loss of CG during heating under various atmospheres. The heating rate was set to 10 °C/min with a gas flow rate of 30 mL/min. The gas types used were high-purity nitrogen and high-purity oxygen. X-ray diffraction analysis (XRD, Rigaku Smartlab 9 kW, Rigaku Corporation, Tokyo, Japan) was performed to observe the phase transitions of CGCs under different temperatures and atmospheres. The test mode was continuous scanning with a step size of 0.02°, using a Cu target, a scanning rate of 10 (°)/min, and a scanning range of 10°~90°. The pore size distribution, pore length distribution, and pore volume fraction of the ceramsite were characterized by mercury intrusion porosimetry (MIP, Autopore IV 9600, Micromeritics Instrument Corp., Norcross, GA, USA). The thermal shrinkage of the CGCs was monitored and recorded in real-time using a heating microscope (HM, Linseis L74, Linseis GmbH, Selb, Germany). The volume density, apparent porosity, closed porosity, and water absorption were directly determined using a ceramic density analyzer with calculations based on the following:

(1) $$P_{a}=\frac{M_{3}-M_{1}}{M_{3}-M_{2}}\times 100\%$$

(2) $$D_{b}=\frac{M_{1}D_{1}}{M_{3}-M_{2}}$$

(3) $$P_{t}=\frac{D_{t}-D_{b}}{D_{t}}\times 100\%$$

(4) $$P_{c}=P_{t}-P_{a}$$

M_1_ = dry weight of sample (g); M_2_ = suspended weight in liquid (g); M_3_ = saturated surface-dry weight (g); P_a_ = apparent porosity (%); P_c_ = closed porosity (%); P_t_ = true porosity (%); D_b_ = bulk density (g/cm^3^); D_t_ = true density (g/cm^3^); D_1_ = immersion liquid density (g/cm^3^), typically water (ρ = 1 g/cm^3^).

## 3. Results and Discussion

### 3.1. Effect of Temperature and Pyrolysis Atmosphere on the Thermal Release of CG

**Figure 2:**
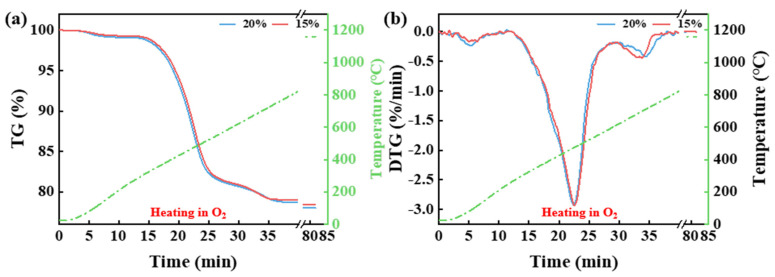
The TG (**a**) and DTG (**b**) curves of CG under different O_2_ concentrations (15% and 20%).

Figure 2 shows the TG and DTG curves of CG under different O_2_ concentrations (15% and 20%). The figure indicates that the O_2_ concentration influences the final weight loss. As the oxygen concentration increases, the maximum weight loss rises from 21.58% to 22%. Furthermore, the combustion of intrinsic moisture, volatile matter, and fixed carbon in CG is affected by the O_2_ concentration. The onset and endpoint of weight loss due to the release of intrinsic moisture, volatiles, and the combustion of fixed carbon occur earlier with an increase in O_2_ concentration. This observation is consistent with the findings of Gu et al. [13]. However, no significant correlation was found between the oxygen concentration and the maximum weight loss rate.

**Figure 3:**
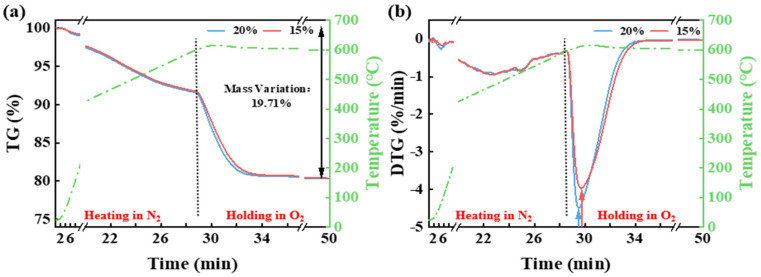
The TG (**a**) and DTG (**b**) curves of CG from room temperature to 600 °C under a N_2_ atmosphere and held at different O_2_ concentrations.

**Figure 4:**
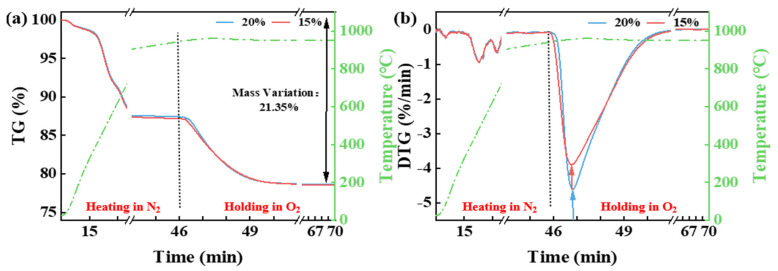
The TG (**a**) and DTG (**b**) curves of CG from room temperature to 950 °C under a N_2_ atmosphere and held at different O_2_ concentrations.

**Figure 5:**
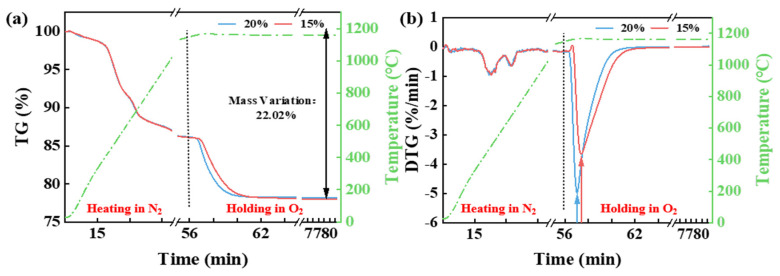
The TG (**a**) and DTG (**b**) curves of CG from room temperature to 1160 °C under a N_2_ atmosphere and held at different O_2_ concentrations.

To examine the impact of different O_2_ concentrations on the thermal release behavior of CG, the heating and cooling were carried out under a N_2_ atmosphere, except during the holding stages at 600 °C, 950 °C, and 1160 °C, where the atmosphere was adjusted to varying O_2_ concentrations. The cooling occurred within the furnace under N_2_. The results are presented in Figure 3, Figure 4 and Figure 5.

Figure 3 shows that during the heating of CG to 600 °C in a N_2_ atmosphere, a weight loss of 8.4% still occurs, mainly due to the release of free water, bound water, and volatile matter. When the temperature reaches 600 °C and different O_2_ concentrations are introduced, the weight loss becomes more pronounced. As the oxygen concentration increases, the maximum weight loss rate rises, and the temperature at which the maximum rate occurs shifts to a lower value. However, the reaction endpoint remains unaffected by the oxygen concentration, with the total weight loss at 600 °C reaching 19.71%.

Figure 4 illustrates that during the heating of coal gangue to 950 °C in a N_2_ atmosphere, a weight loss of 12.61% is observed, once again due to the release of free water, bound water, and volatile matter. When the temperature reaches 950 °C and different O_2_ concentrations are introduced, significant weight loss continues. However, the temperature corresponding to the maximum weight loss rate at this stage is independent of the O_2_ concentration. With a higher O_2_ concentration, the maximum weight loss rate increases. The reaction endpoint, similar to the case at 600 °C, is unaffected by the O_2_ concentration, and the total weight loss at 950 °C is 21.35%.

Figure 5 reveals that during heating to 1160 °C in a N_2_ atmosphere, a weight loss of 13.94% occurs due to the release of free water, bound water, and volatile matter. When the temperature reaches 1160 °C and different O_2_ concentrations are introduced, coal gangue continues to lose weight. At this temperature, the maximum weight loss rate occurs earlier as the O_2_ concentration increases. Additionally, the higher the O_2_ concentration, the faster the maximum weight loss rate. Unlike the previous temperatures, the reaction endpoint at 1160 °C is influenced by the oxygen concentration. The final weight loss at 1160 °C is 21.82% with 20% O_2_ and 22.02% with 15% O_2_.

### 3.2. Phase Transformation and Shrinkage During the Sintering Process of CGCs

**Figure 6:**
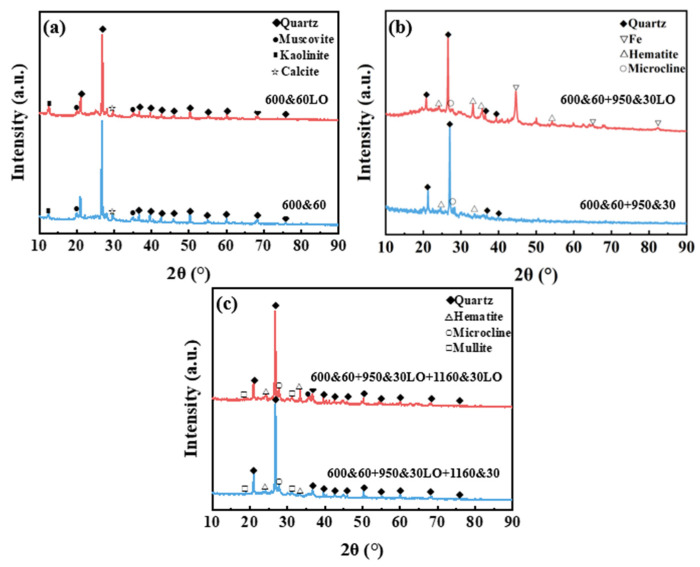
XRD patterns of CGCs sintered at different temperatures and oxygen concentrations: 600 °C (**a**), 950 °C (**b**), and 1160 °C (**c**).

The XRD patterns of CGCs at different temperatures and O_2_ concentrations are shown in Figure 6. At 600 °C, the main crystalline phases—quartz, muscovite, kaolinite, and calcite—are unaffected by the atmosphere. At 950 °C, the phases differ based on the O_2_ concentration; CGCs sintered in 20% O_2_ show quartz, microcline, and hematite, while in 15% O_2_, elemental iron also appears. At 1160 °C, the main phases, including quartz, hematite, microcline, and mullite, remain the same regardless of the atmosphere.

**Figure 7:**
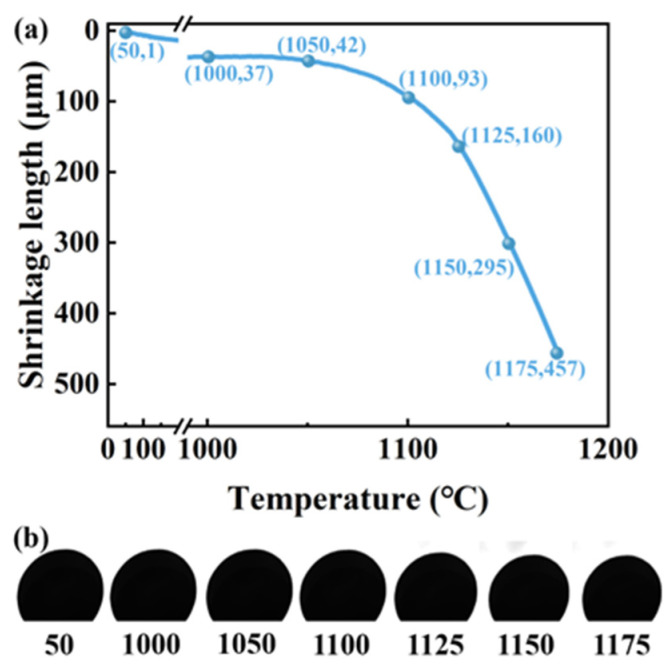
The macroscopic shrinkage dimensions during the sintering process of a CGC (**a**) and real-time recorded images (**b**).

The macroscopic dimensional changes of a CGC during sintering from room temperature to 1170 °C are shown in Figure 7. Figure 7a presents the shrinkage curve of an 8 mm diameter CGC sintered at a rate of 5 °C/min in a 20% O_2_ atmosphere, reaching 1175 °C. The shrinkage from room temperature to 1000 °C was minimal, measuring only 37 μm with a shrinkage rate of 0.46%. Above 1050 °C, the shrinkage intensified, with the fastest rate observed beyond 1100 °C. Figure 7b provides real-time images of the ceramsite at 50, 1000, 1050, 1100, 1125, 1150, and 1175 °C, clearly illustrating the macroscopic shrinkage on the surface.

**Figure 8:**
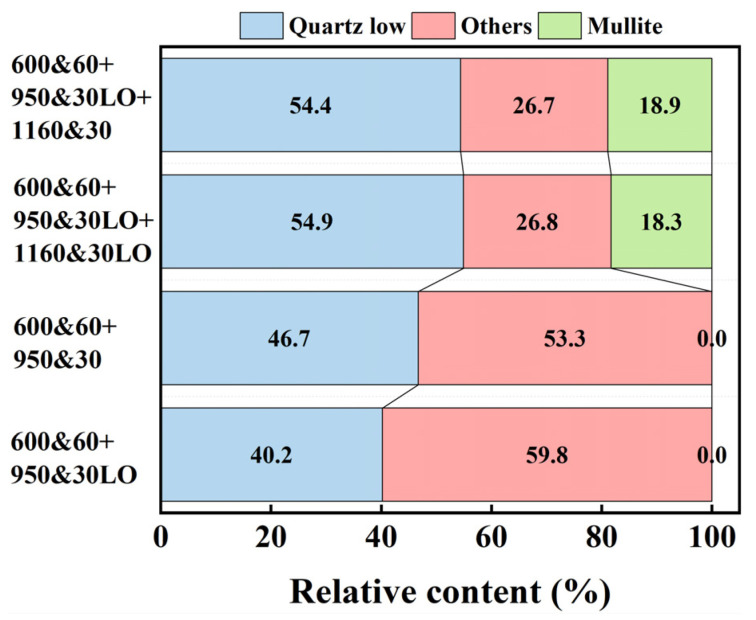
The relative content of the dominant crystalline phase.

A Rietveld quantitative analysis of the main crystalline phases was performed on the XRD patterns of the coal gangue ceramsite sintered under four regimes, and the results are shown in Figure 8 [14,15,16]. The formation of mullite is accompanied by the generation of micropores [17]. The occurrence of micropores indicates an increase in the shrinkage of the ceramsite, which further enhances the strength of the ceramsite. The relative content of the mullite phase increases with the rise of the temperature, and the relative content of the mullite phase changes little after 1160 °C, which further indicates that the main crystalline phase of coal gangue ceramsite at 1160 °C is independent of the atmosphere. The shrinkage of ceramsite is affected by two factors. One is caused by the occupation of internal pores by ion diffusion and densification during sintering, which is manifested as a decrease in the porosity. The other is the pore shrinkage during the chemical transformation. For example, the density of mullite is 3.16 g/cm^3^, while the true density of coal gangue powder after calcination at 1000 °C is 2.83 g/cm^3^. Therefore, the precipitation of mullite leads to an increase in the component density and a decrease in the volume, thereby improving the strength of coal gangue ceramsites. 

### 3.3. Effect of Temperature Regime and Pyrolysis Atmosphere on the Pore Structure of CGCs

**Figure 9:**
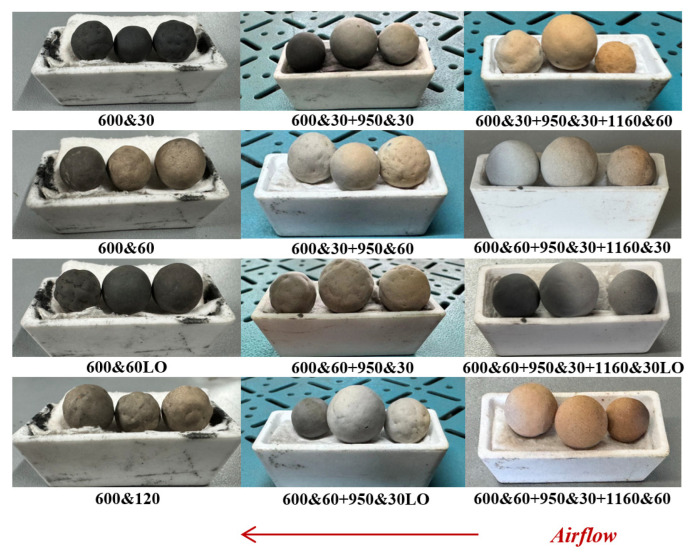
CGCs under the action of different atmospheres and temperature regimes.

Figure 9 presents the appearance of CGCs sintered at various temperatures under different pyrolysis atmospheres. As the temperature increases, the CGCs gradually lighten in color and turn yellow. At 600 °C, after 30 min of holding in a 20% O_2_ atmosphere, the CGCs appear slightly darker than the raw material due to pyrolysis and macromolecular decomposition. With a prolonged holding time, they first lighten and then turn yellow, with the regions exposed to oxygen showing more distinct color changes. The same color change pattern is observed with CGCs at 950 °C. At 1160 °C, extended exposure leads the CGCs to lighten, turn yellow, and eventually form a hardened outer shell due to densification. The atmosphere primarily influences the transition from black to yellow.

**Figure 10:**
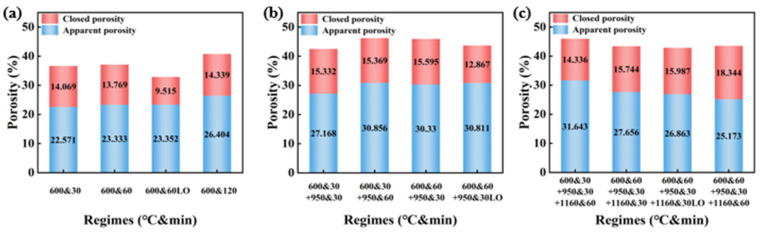
Porosity of CGCs under varying atmospheres and temperature conditions: 600 °C (**a**), 950 °C (**b**), and 1160 °C (**c**).

Figure 10 illustrates the porosity changes of CGCs under varying temperatures and pyrolysis atmospheres. At 600–950 °C, with a constant atmosphere, the oxidation time is uniform across the temperatures, resulting in similar pore structures. An increased oxidation time raises apparent porosity while closed porosity remains unchanged. In an oxidative atmosphere, apparent porosity remains constant, but closed porosity increases. At 950–1160 °C, with a constant atmosphere, apparent porosity depends on the oxidation time below 950 °C, increasing with longer oxidation time, while closed porosity remains nearly constant. In this temperature range, a longer oxidation time reduces apparent porosity and increases closed porosity, while a more oxidative atmosphere increases apparent porosity with unchanged closed porosity.

**Figure 11:**
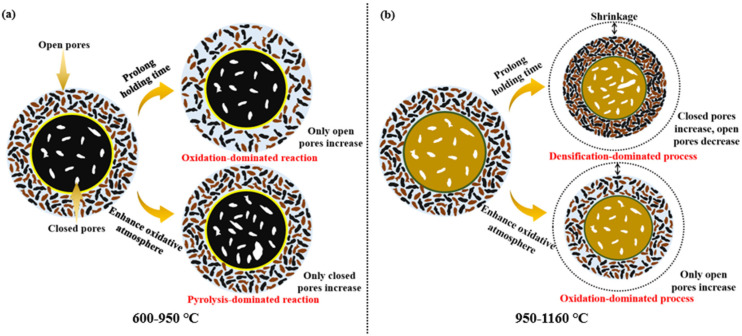
Mechanism of Apparent and closed pore variation of CGCs under different atmospheres and temperature regimes: 600–950 °C (**a**) and 950–1160 °C (**b**).

Based on the thermal release characteristics of CG under different temperatures and pyrolysis atmospheres shown in Section 3.1, one reason for the differences in the pore structures of CGCs is the oxidation and pyrolysis reactions. At 600–950 °C, with a constant atmosphere, increasing the holding time raises apparent porosity because oxidation dominates this stage, with apparent pores forming mainly due to the weight loss from oxidation reactions. Continuous oxidation on the CGC surface enlarges apparent pores. With the same total oxidation time across the different temperatures, the pore structures are similar because closed pore formation is primarily associated with the rate of pyrolysis, which is independent of the oxidation time. When the atmosphere is more oxidative, closed porosity increases because the formation of closed pores is linked to the pyrolysis process. An increased oxygen concentration promotes pyrolysis, leading to more gaseous, liquid, and solid products [18,19,20,21,22]. Secondary reactions occur between pyrolysis vapors and O_2_. Increasing the O_2_ content enhances the pyrolysis reaction, which accelerates the release of volatile components, enlarging the closed pores and increasing the amount of pores [23,24]. Additionally, pyrolysis consumes micropores and mesopores and causes the merging of adjacent pores, contributing to an increased closed porosity. Figure 11a illustrates the mechanism of the apparent and closed pore variation of CGCs under different atmospheres and temperature regimes from 600–950 °C.

At 950–1160 °C, with a constant atmosphere, extending the holding time decreases apparent porosity and increases closed porosity. This is due to the densification process causing CGCs to shrink and reduce apparent porosity. The formation of mullite creates density differences inside the CGC, leading to pore shrinkage and increased closed porosity. In an oxidative atmosphere, closed porosity remains unchanged while apparent porosity increases slightly due to minor oxidation-related weight loss during the densification process. The CO produced from pyrolysis is further oxidized to CO_2_, and the CO_2_ is reduced to CO at high temperatures, which changes the apparent porosity of the ceramsite. Closed porosity remains unaffected because pyrolysis reactions nearly stop above 1000 °C, reducing weight loss, which aligns with the related research [25]. At temperatures above 1000 °C, volatile products from pyrolysis decrease sharply, but carbon oxidation continues, corresponding to the observed increase in apparent porosity and unchanged closed porosity. Figure 11b illustrates the mechanism of the apparent and closed pore variation of CGCs under different atmospheres and temperature regimes from 950–1160 °C.

For the temperature range of 600–950 °C, with the same total oxidation time, extending the oxidation time in the 950–1160 °C range reduces apparent porosity and increases closed porosity. This is because ceramsite with sufficient oxidation at low temperatures mainly experiences effects from densification and mullite formation at high temperatures. The extended holding time exacerbates shrinkage and increases the mullite content. In the 950–1160 °C range, apparent porosity depends on the oxidation time below 950 °C. Longer oxidation times result in less noticeable increases in apparent porosity and nearly unchanged closed porosity. This is because ceramsite with sufficient oxidation loses less weight in the 950–1160 °C range, with densification reducing apparent porosity, while ceramsite with shorter oxidation times continues to oxidize and lose weight, leading to increased apparent porosity.

### 3.4. Moisture Retention and Water Storage Characteristics of Porous Ceramsites with Different Sizes

**Figure 12:**
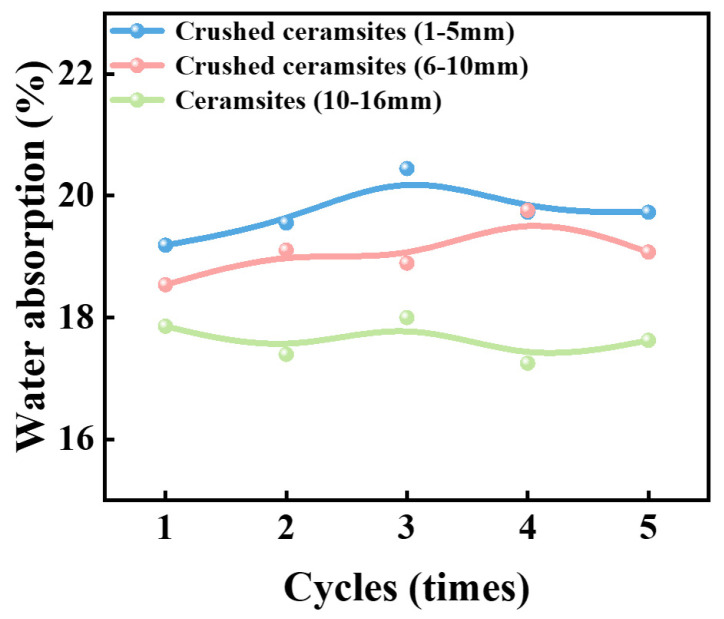
Moisture retention, water storage properties, and dry–wet cycle characteristics of moisture-retaining materials with different sizes.

Batches of ceramsite samples fired under the 600&30 + 950&60 system were subjected to classification and crushing processing, yielding three types of moisture-retaining materials with particle sizes ranging from 1–5 mm, 5–10 mm, and 10–16 mm. The water storage and dry–wet cycle characteristics of the samples were studied, and the results are shown in Figure 12.

The water absorption capacity of the three size-specific moisture-retaining materials follows the following order: ceramsites with a particle size of 1–5 mm, ceramsites with a particle size of 6–10 mm, and ceramsites with a particle size of 10–16 mm. All of the samples have a stable pore structure, for after 5 dry–wet cycles, no significant change was observed in the water storage capacity of the three moisture-retaining materials.

**Figure 13:**
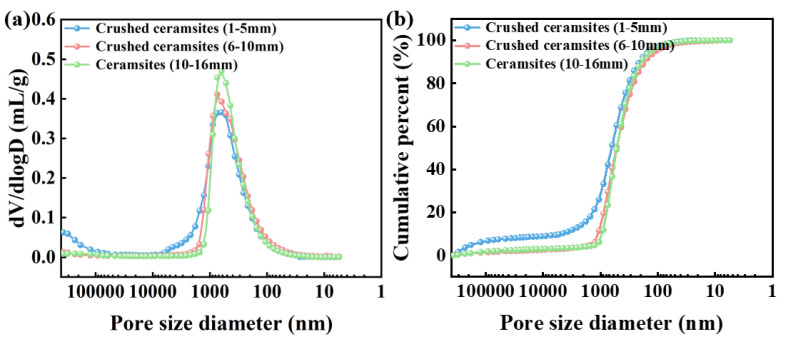
Pore size distribution (**a**) and corresponding pore volume proportion (**b**) of moisture-retaining materials with different shapes.

The pore size distribution of moisture-retaining materials with different sizes was tested by MIP and is shown in Figure 13. It can be seen that the 1–5 mm ceramsites have a higher proportion of pore volume above 1000 nm, followed by the 6–10 mm ceramsites, and finally the 10–16 mm ceramsites. This indicates that the crushing process destroys the micropores with a diameter of less than 1000 nm. The water storage performance of moisture-retaining materials is mainly related to the volume of macropores above 1000 nm, as these macropores can effectively store water.

## 4. Conclusions

The residual coal in coal gangue ceramsites (CGCs) has a more complex pore structure than traditional clay ceramsites during the sintering process. Under varying temperatures and atmospheres, the pore structure of CGCs is influenced by oxidation and pyrolysis reactions, phase transformations, and densification shrinkage.

At 600–950 °C, with a constant atmosphere, the holding time primarily affects apparent porosity, as apparent pores mainly form due to weight loss from oxidation. An increased oxidative atmosphere raises closed porosity, with closed pores forming due to the pyrolysis process. Higher oxygen concentrations accelerate pyrolysis, increasing closed porosity through reactions, diffusion, and pore merging.

At 950–1160 °C, the holding time affects both apparent and closed porosity due to densification shrinkage and mullite formation. In an oxidative atmosphere, apparent porosity increases because pyrolysis nearly ceases above 1000 °C, but carbon oxidation continues, impacting the pore structure.

The porous ceramsites prepared by this sintering system exhibit excellent dry–wet cycle characteristics, with no significant decrease in their water storage capacity after five cycles of repeated water absorption and drying. The water storage performance of the ceramsites is mainly related to the volume of macropores above the micrometer scale, as these macropores can effectively store water.

In subsequent experimental studies on the pore structure regulation mechanisms of coal gangue ceramsites, apparent and closed pores can be tailored separately to achieve the precise structural control of the pores, thereby broadening their application spectrum in absorption, insulation, and other uses.

## Figures and Tables

**Table 1:** Chemical compositions of coal gangue (CG) (wt.%) [12].

Temp.	SiO_2_	Al_2_O_3_	Fe_2_O_3_	CaO	MgO	S	K_2_O	TiO_2_	Na_2_O	LOI	Total
25 °C	55.14	20.10	5.26	0.76	1.59	0.40	2.57	0.79	1.24	11.76	99.65
1150 °C	59.67	20.78	6.72	2.00	1.50	0.43	2.90	0.82	1.58	2.91	99.33

LOI: Loss on ignition.

**Table 2:** Results of proximate analysis and calorific value of CG [12].

Proximate Analysis (wt.%)					*Q*_net,ar_ (Kcal/kg)	
*M*_t_	*M*_ad_	*A*_ad_	*V*_ad_	*FC*_ad_	573	
2.7	0.66	84.86	9.57	4.91		

*M*_t_—total moisture; *M*_ad_—moisture on air dry basis; *A*_ad_—ash on air dry basis; *V*_ad_—volatile on air dry basis; *FC*_ad_—fixed carbon on air dry basis; *Q*_net,ar_—low calorific value.

## Data Availability

The original contributions presented in this study are included in the article. Further inquiries can be directed to the corresponding author.
